# CARbon DIoxide for the treatment of Febrile seizures: rationale, feasibility, and design of the CARDIF-study

**DOI:** 10.1186/1479-5876-11-157

**Published:** 2013-06-27

**Authors:** Stephanie Ohlraun, Tobias Wollersheim, Claudia Weiß, Peter Martus, Steffen Weber-Carstens, Dietmar Schmitz, Markus Schuelke

**Affiliations:** 1 NeuroCure Clinical Research Center, Charité Universitätsmedizin Berlin, Berlin, Germany; 2 Department of Anesthesiology and Operative Intensive Care Medicine, Charité Universitätsmedizin Berlin, Berlin, Germany; 3 Department of Neuropediatrics, Charité Universitätsmedizin Berlin, Berlin, Germany; 4 Neuroscience Research Center, Charité Universitätsmedizin Berlin, Berlin, Germany; 5 Bernstein Center for Computational Neuroscience, Charité Universitätsmedizin Berlin, Berlin, Germany; 6 Institute for Clinical Epidemiology and Applied Biostatistics, Eberhard-Karls-University, Tübingen, Germany

**Keywords:** Febrile Seizure, Alkalosis, Carbogen, Carbon Dioxide, Clinical Trial, Intervention, Translation

## Abstract

**Background:**

2-8% of all children aged between 6 months and 5 years have febrile seizures. Often these seizures cease spontaneously, however depending on different national guidelines, 20-40% of the patients would need therapeutic intervention. For seizures longer than 3-5 minutes application of rectal diazepam, buccal midazolam or sublingual lorazepam is recommended. Benzodiazepines may be ineffective in some patients or cause prolonged sedation and fatigue. Preclinical investigations in a rat model provided evidence that febrile seizures may be triggered by respiratory alkalosis, which was subsequently confirmed by a retrospective clinical observation. Further, individual therapeutic interventions demonstrated that a pCO_2_-elevation *via* re-breathing or inhalation of 5% CO_2_ instantly stopped the febrile seizures. Here, we present the protocol for an interventional clinical trial to test the hypothesis that the application of 5% CO_2_ is effective and safe to suppress febrile seizures in children.

**Methods:**

The CARDIF (**CAR**bon **DI**oxide against **F**ebrile seizures) trial is a monocentric, prospective, double-blind, placebo-controlled, randomized study. A total of 288 patients with a life history of at least one febrile seizure will be randomized to receive either carbogen (5% CO_2_ plus 95% O_2_) or placebo (100% O_2_). As recurrences of febrile seizures mainly occur at home, the study medication will be administered by the parents through a low-pressure can fitted with a respiratory mask. The primary outcome measure is the efficacy of carbogen to interrupt febrile seizures. As secondary outcome parameters we assess safety, practicability to use the can, quality of life, contentedness, anxiousness and mobility of the parents.

**Prospect:**

The CARDIF trial has the potential to develop a new therapy for the suppression of febrile seizures by redressing the normal physiological state. This would offer an alternative to the currently suggested treatment with benzodiazepines. This study is an example of academic translational research from the study of animal physiology to a new therapy.

**Trial registration:**

ClinicalTrials.gov identifier: NCT01370044

## Background

Febrile seizures are defined as cerebral seizures occurring in children between 6 months and 5 years of age that are solely associated with fever and not with CNS infection or previous neonatal or unprovoked seizures, and do not meet defining criteria for acute symptomatic seizures [1]. Febrile seizures are the most common type of cerebral seizures in childhood and affect between 2-8 % of all children, depending on geographical and ethnic factors, as well as on ascertainment definitions and methods [2-4]. The peak incidence of febrile seizures is around 16-18 months-of-age [5-7]. Regarding the duration of febrile seizures, Hesdorffer *et al.* investigated 158 children with a first febrile seizure and distinguished two groups, one with an average duration of 3.8 minutes (82% of the study population) and a second one with 39.8 minutes (18%). In 38-54% of the children the duration of febrile seizure was above 5 and 3 minutes, respectively, and would thus have triggered pharmacological intervention according to the guidelines [8-10]. In about 30% of affected children febrile seizures are recurrent [11]. Most febrile seizures have a benign prognosis, but their occurrence during childhood is associated with an increased risk of 2.4-8.0% to develop epilepsy later in life [12,13]. In contrast to simple febrile seizures, prolonged febrile seizures or febrile status epilepticus (seizure duration > 30 min) have a substantial higher risk to cause short- and long-term brain abnormalities. For example, it has been shown that complex febrile seizures are associated with an increased T_2_-signal intensity on cranial MRI, most likely reflecting acute injury of the hippocampal formation [14,15]. “Simple” febrile seizures mainly occur within the first 24 hours of a febrile illness and only once during a fever period, last less than 15 minutes and do not show a focal component. “Complex” febrile seizures last longer than 15 minutes, occur several times during one fever episode, and may have focal features. The duration of recurrent febrile seizures is often longer than that of single ones [11].

In Germany, no commonly agreed and published guidelines exist regarding the treatment of febrile seizures, but a published Italian guideline recommends waiting for the febrile seizure to cease spontaneously within the first 3 minutes. Only after this “waiting period” benzodiazepines (rectal diazepam, buccal midazolam, or sublingual lorazepam) should be administered as the “standard therapy”, which will reach pharmacologically active concentrations in the CNS after 3-5 minutes [9]. However, benzodiazepines are effective in only 60-80% of children with seizures [16,17]. Furthermore, due to its sedative effect, children may sleep for the rest of the day. Undesirable strong sedation and fatigue, headache, ataxia, confusion, anterograde amnesia, and paradoxical stimulatory effects are potential side effects of benzodiazepines. The incidence of respiratory depression after the rectal application of diazepam is 5-8% with some patients even requiring mechanical ventilation [18-20]. Additionally, the sedative effect may impede a precise pediatric assessment and possibly lead to unnecessary invasive diagnostic procedures such as a lumbar puncture. As children with febrile seizures are mainly treated at home, these potential side effects should be taken into consideration and an alternative to the currently used pharmacological therapy would be welcome.

Schuchmann *et al.* reported an animal model, in which they raised to 40-41°C the body temperature of young rats aged P8-11 (corresponding to a human developmental age of 1-5 years) and P22-23 (≈ human 12-18 years) in a heated chamber [21]. Only all the young rats (P8-11, n = 29) had cerebral seizures, but none of the older ones. The authors demonstrated a disproportional increase of the breathing frequency (hyperventilation) in young rats along with the rise of body temperature. Hyperventilation led to hypocapnia, subsequent respiratory alkalosis, elevation of the intracranial pH, and thus an increase of the excitability of cortical neurons. To restore the normal physiological state, the authors increased the ambient CO_2_ concentration to 5%. Indeed, the blood pCO_2_ normalized and the seizures stopped within 15-20 seconds, thus verifying the antiepileptic effect of CO_2_ under these conditions.

These findings prompted us to investigate, whether febrile seizures might also be associated with and triggered by respiratory alkalosis in human patients. In a retrospective study Schuchmann *et al.* analyzed the blood gas measurements of 433 children who were admitted to the pediatric emergency department of the Charité with various conditions. They discovered that up to 2 hours after the febrile seizure the blood pH in children was significantly higher and the pCO_2_ levels lower than in children admitted for other reasons (e.g. gastroenteritis, upper respiratory tract infection) [22]. Beyond that, individual uncontrolled therapeutic interventions have been reported, by which seizures could be stopped through elevation of the bood-pCO_2_ either *via* re-breathing [23] or by carbogen inhalation [24]. Thus, there is preclinical evidence from experimental febrile seizures and case reports supporting the idea that fever-related respiratory alkalosis plays a role in the generation of febrile seizures [25]. These findings and the uncritical safety profile of carbogen, which has been used for long in various diagnostic procedures, encouraged us to translate these findings into a controlled interventional clinical trial in a domestic setting that combines phases I and II – the CARDIF study.

## Methods

### Study objective

We hypothesize that carbogen administered through a low-pressure can fitted with a respiratory mask is safe and interrupts recurrences of febrile seizure more effectively than placebo (pure oxygen) within 3 minutes.

### Application device

**Figure 1:**
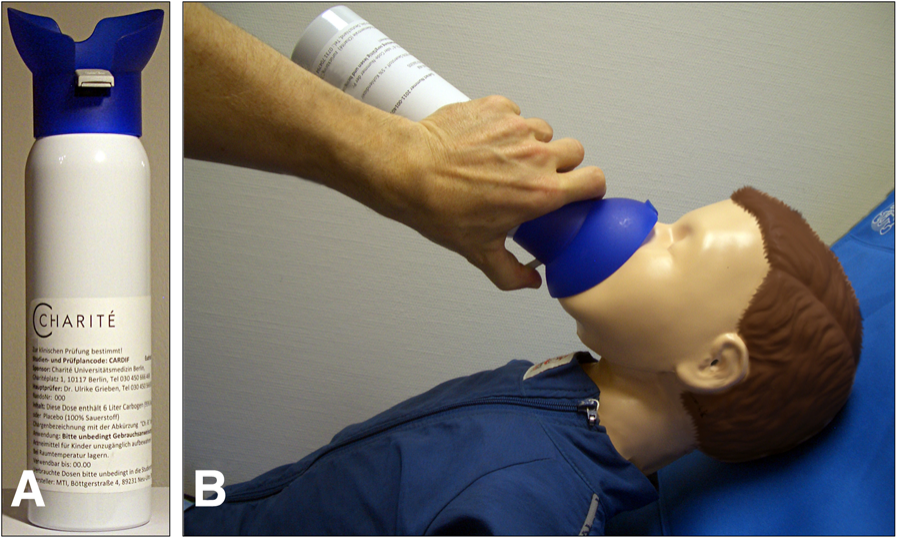
**The application device. (A)** Low-pressure can containing 6 liter of the study medication with attached, loosely fitting, “one-size for all” respiratory mask. Pressing on the grey cantilever initiates the gas flow. **(B)** Application of the study medication to a child. The parents are advised to press on the grey cantilever until the audible gas-flow has stopped (after ≈3 minutes).

**Figure 2:**
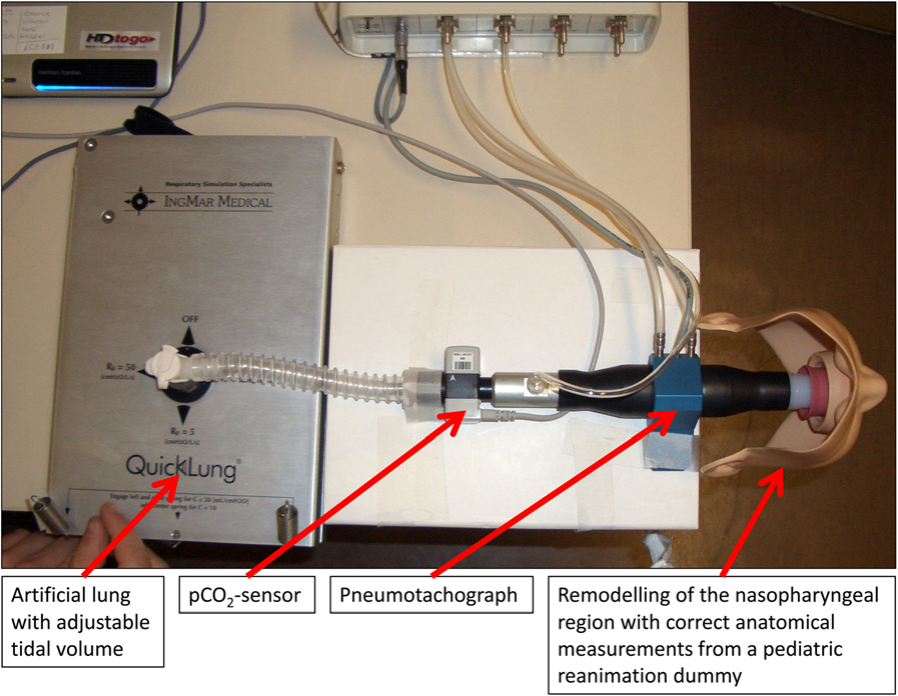
**Experimental setting.** Assessment of inspiratory & endtidal pCO_2_, respiratory volume & rates, air flow velocity & pressure was done using an artificial lung, a pCO_2_-sensor, a pneumotychograph, and an anatomical facsimile of a face with nasopharynx and oral cavity of a 2 year-old child. Accordingly, anatomical dead space was adjusted to 40 ml.

**Figure 3:**
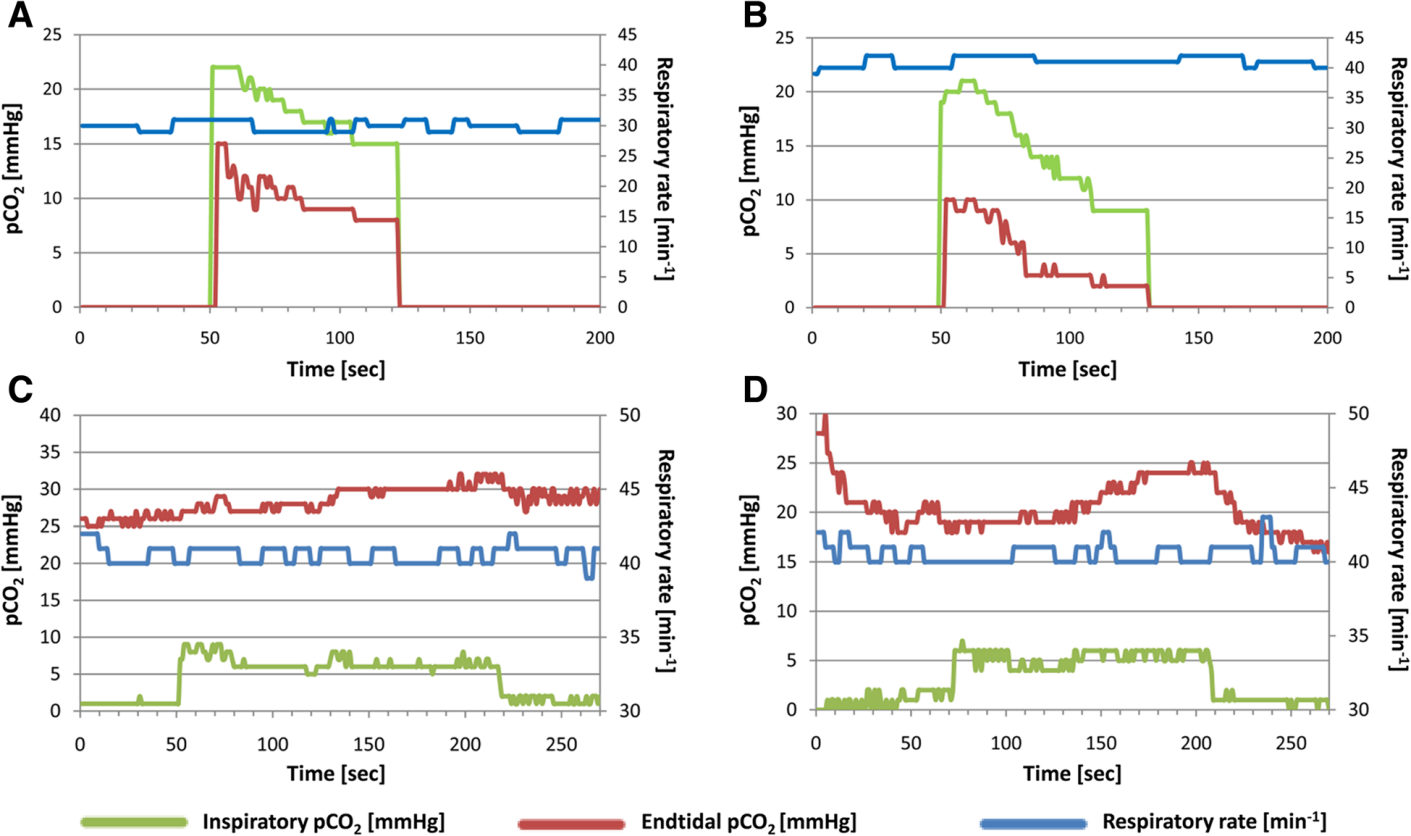
**Inspiratory and endtidal pCO**_**2 **_**concentrations. (A-B)** Course of the inspiratory and endtidal pCO_2_ using the experimental setting described in Figure 2. The gas flow was started after 50 seconds and continued for 60 seconds. The experiment was repeated with two different respiratory rates and tidal volumes: **(A)** 30/min | 90 ml (corresponding to 2-year-old child) and **(B)** 40/min | 65 ml (corresponding to a 9-months-old infant). In both cases we observed an increase of the inspiratory pCO_2_ by a maximum of ≈20 mmHg. **(C-D)***In vivo* respiration of 6 liter CO_2_ over 3 minutes: the gas flow was again started at 50 seconds. The panels depict two different experimental conditions: **(C)** The test person tried to maintain a respiratory rate of 40/min in synchrony with a metronome over the entire period. Under these conditions the endtidal pCO_2_ rose by 7 mmHg. In setting **(D)** the test person had hyperventilated before the start of the gas flow. Starting from a lower pCO_2_ of 17 mmHg, the pCO_2_ again rose by 7 mmHg.

In order to verify that inhalation of 6 liters carbogen over 3 minutes through a loosely fitting mask from a low-pressure can (Figure 1) achieves the required increase of endtidal pCO_2_, we tested the device in a respiratory model and in a human adult volunteer (Figures 2 &3). The respiratory model was composed of an artificial test lung (QuickLung®, IngMar Medical, Pittsburgh, PA, USA), airways with adjusted dead space, a pneumotachograph (Metabo, Epalinges, Switzerland), sidestream capnography (Microstream® and IntelliVue® MP30, Philips, Hamburg, Germany) and an anatomical model of the infantile nasopharynx. The tidal volume, dead space and the respiratory frequency were set to several age-specific physiological values. Breaths were generated by manually pumping the test lung. During the investigations predefined tidal-volumes were kept constant by fixing the hub of the test lung to a certain volume. Frequency was given by a metronome. Resulting respiratory minute-volumes, breathing rates, air flow, airway pressure and pCO_2_ before, during and after carbogen inflation were continuously recorded with a sampling rate of 50 Hz (*ICU*-*Lab*, *KleisTEK* Engineering, Bari, Italy and ICM^+^, University of Cambridge, England). The *in vivo* test was done with a healthy adult volunteer, whose endtidal pCO_2_ was continuously measured with same sidestream pCO_2_ device as before, during and after carbogen inflation. Finally, in order to verify that breathing from the mask, which always would imply some degree of re-breathing, did not unduly increase the endtidal pCO_2_, the volunteer breathed into the mask with stopped gas flow while inspiratory and endtidal pCO_2_ as well as SaO_2_ were simultaneously measured.

### Study design

**Figure 4:**
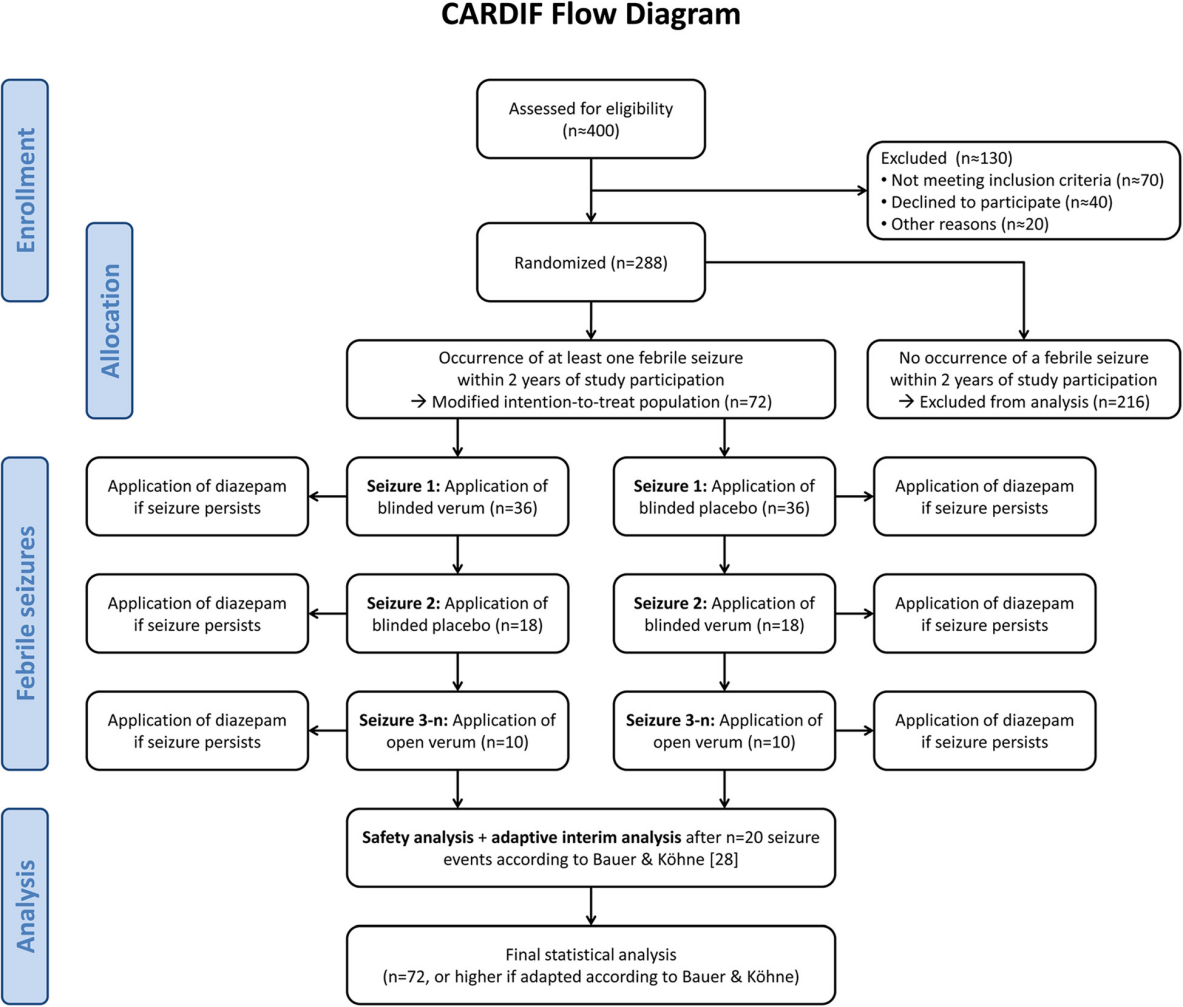
Flow diagram of the CARDIF-trial.

The CARDIF study is a monocentric, prospective, double-blind, placebo-controlled, randomized study. It has been approved by the Ethical Review Board of the State of Berlin and the Competent Regulatory Authority, and is registered at the U.S. Clinical Trials Registration System (NCT01370044). The detailed study design according to the revised CONSORT statement [26,27] is described below (see also Figure 4). The study design had to meet the following challenges:

#### Route and method of CO_2_ application

As febrile seizures usually occur at home, safety and practicability aspects had to be specifically addressed while selecting the appropriate method for CO_2_ delivery. The pCO_2_ in human blood may be increased by two methods: one is to re-breathe exhaled air through a plastic bag (the re-breathing technique), which is commonly used to treat acute hyperventilation syndrome and panic disorders [25,28]. In young infants, however, this technique requires special expertise as prolonged re-breathing might reduce inspiratory oxygen concentration and induce hypoxia. Re-breathing must thus not be applied without the possibility to measure endtidal pO_2_ and pCO_2_[23]. A second, much safer method is the application of CO_2_-enriched air or of medical carbogen (5% CO_2_ plus 95% O_2_), which ensures that the blood pCO_2_ does not increase and the pO_2_ does not drop beyond certain limits. The added oxygen even improves oxygenation and prevents hypoxia. As febrile seizures usually occur at home, where no blood gas monitoring is possible, we opted for carbogen to be used in our clinical trial. For this procedure we developed the concept of a special low-pressure can with an attached mask (Figure 1). The pressurized gas secures high enough flow rates to obtain the desired intra-tracheal pCO_2_ (Figure 3).

#### Biometric design

As the preexisting clinical evidence was limited, we chose a two phase adaptive design according to Bauer & Köhne [29], which comprises an adaptive interim analysis to allow for sample size modifications without compromising the overall significance level to assure the overall power of the study.

#### Randomization

Due to procedural constraints, the randomization cannot take place while the child has a seizure, but has to be done immediately after inclusion into the study. As there is only a 25-30% chance that the included patients will indeed have a febrile seizure recurrence during the 24 months study period, thrice as many individuals have to be randomized than will be finally included into the analysis. The individuals for the analysis will thus be chosen according to the principle of the “modified intention-to-treat population” [30]. Accordingly, only patients with a febrile seizure recurrence will be analyzed in the intention-to-treat analysis. A total of 288 patients will be included into the study, 80 for the first and 208 for the second study phase. We expect to include 20+52 = 72 patients with febrile seizure recurrences into the final analysis. This number could be corrected upwards after completion of the first phase, but not downwards (see *Statistical analysis*).

#### “*Crossover” design*

After the first seizure recurrence a crossover occurs. With this, regardless of the result of the first treatment, patients who received placebo for the first seizure recurrence receive verum for the second one or *vice versa*. From the third seizure recurrence onwards, all patients receive open label verum. Since it is anticipated that only a minority of patients will suffer from a second seizure recurrence and thus enter the crossover arm, the study is not a true crossover study. Data from the “crossover” and the open label extension phase will thus only be considered for secondary analyses. The primary analysis will only include the first seizure recurrence, while the secondary analyses consider all seizure recurrences per patient.

### Primary and secondary outcome

The primary outcome is the efficacy of a 3 minute application of carbogen to stop a febrile seizure recurrence. Secondary outcome parameters are safety, practicability to use the can, quality of life, contentedness, and anxiousness and mobility of the parents.

Treatment failure is defined as a febrile seizure recurrence, which has been treated with the study medication but did not cease within the first 3 minutes, so that the “standard therapy” (rectal diazepam) has to be applied. Safety is defined as the number of adverse events. The practicability to handle the can and mask are assessed by a questionnaire. Further, parents are asked to report on their quality-of-life, contentedness, anxiousness and mobility. These data are gathered in an open interview following standardized study-related questions.

### Patient population

Patients will be recruited from the Children’s University Hospital of the Charité in Berlin. Parents or custodians of potential participants will be thoroughly informed about the study rationale, procedures, potential risks and benefits. After written informed consent, children will be included into the study if all study selection criteria (see Table 1) are met. The study begins with the randomization into one of the two arms. Parents are trained hands-on by the study personnel to assemble the mask and use the can (Figure 1). This training is complemented by written instructions.

**Table 1:** In - and exclusion criteria

**Inclusion criteria**	**Exclusion criteria**
1. Written informed consent from the parents or custodians of the study participant.	1. Suspected or proven meningitis or encephalitis as a possible cause for a past cerebral seizure associated with fever.
2. At least one febrile seizure in the past medical history of the study participant.	2. Presence of a neurological disease or a brain malformation.
3. Age 12 months—5 years in phase 1 and age 6 months—5 years in phase 2 of the adaptive design (after observation of 20 seizures, approximately patients number 81-288).	4. Cerebral seizures without fever in the past medical history.
	5. Proof of spike-wave discharges or of a focus in the in the interval EEG.
	6. Presence of a respiratory illness (e.g. bronchial asthma or bronchopulmonary dysplasia, BPD).
	7. Presence of other severe organ or chronic diseases.
	8. Current participation in another clinical trial.
	9. Failure to provide written informed consent on part of the parents or custodians with regard to the storage and dissemination of pseudonymized study results.
	10. Known adverse reactions against carbogen.
	11. Placement of either the child or a parent/custodian in an institution, based on judicial orders.
	12. Lack of understanding of the study procedures on part of the parents or custodians (e.g. lack of language knowledge or of general intellectual capacity).

### Study medication

Carbogen is a gas mixture composed of 5% CO_2_ and 95% O_2_, which is stored in compressed gas cylinders (Figure 1A). These low-pressure cans are manufactured and filled by the company MTI Industriegase AG (in cooperation with the Westphalen AG, Germany) and contain 6 liters carbogen gas. A breathing mask with attached application cantilever and a folded instruction sheet are shrink-wrapped in plastic foil together with the can. The amount of gas and the size of the valve are adjusted to allow an easily audible gas flow of 3 minutes duration. It is important that the parents realize when the can is empty in order to apply rectal diazepam (“standard therapy”) if the seizure recurrence has not stopped within these 3 minutes of gas application. Carbogen is known to be tolerated very well with only little side effects. Some discomfort has been reported by patients breathing 5% CO_2_ for a prolonged period of time [31,32], which was also seen in healthy volunteers [33]. However, due to the short application time, effects on normal brain function and other side effects are expected to be minimal. Patients suffering from febrile seizures tend to hyperventilate during fever, which leads to a pathological reduction of pCO_2_ in the blood (respiratory alkalosis) [22]. In these patients, carbogen inhalation would thus not lead to a pathological pCO_2_ increase, but restore the physiological normal state. Therefore, the occurrence of side effects is even less likely in patients with febrile seizures than in healthy controls.

In addition to 5% carbon dioxide, carbogen gas contains 95% oxygen. The placebo contains 100% oxygen. Oxygen might in principle have a toxic effect due to the formation of reactive oxygen species (ROS). It should be noted, however, that the application in the study is very brief and not hyperbaric. In patients with febrile seizures, oxygen saturation (SaO_2_) may drop below 90% during a seizure because of irregular breathing. The additional administration of oxygen would improve the SaO_2_ and would be in line with the guidelines [9].

### Randomization and treatment allocation

After inclusion into the study, patients will receive a running patient number. For each patient we reserve a package containing two blinded cans (“A” and “B”). Block randomization will be done by issuing to the parents the first blinded “A” can (randomly containing either verum or placebo) out of consecutively numbered packages. After the first seizure recurrence the parents are issued the “B” can of their package, which contains verum if “A” was placebo and *vice versa*. After a second seizure recurrence the parents will receive further open-label cans that all contain verum. The study duration will be 24 months for each patient. Patients who have at least one seizure recurrence during this time will be included into the (modified) intention-to-treat analysis.

### Study procedures

At study entry all patients receive a thorough physical examination. Neurological assessment includes vigilance, muscle strength and tone, coordination, sensibility, walking performance und cranial nerve status. Parents are instructed to call the study team as soon as the seizure had occurred. Hence a study physician “on call” can be reached round the clock 24/7 by mobile phone, who will then explain and ask a set of standardized questions about the seizure. Parents are asked about the circumstances and the details of the seizure recurrence including the date and time, duration, focality, body temperature at the time of the seizure, cause of the febrile infection and maximum temperature during the entire febrile period. Questions regarding the endpoint comprise: **(i)** cessation of the seizure during the 3 minute application of the study medication, or **(ii)** the necessity to use diazepam thereafter. Further questions are related to side effects, ratings on the practicability, quality of life, anxiousness, contentedness and mobility with regard to the use of the can. Parents are further asked to present to the study center as soon as possible to exchange the study medication. If the family cannot come to the study center, a study physician visits the family at home. If parents do not contact the study center for 6 months, the study team will phone the family to make sure that no seizure recurrence had occurred in the meantime and to inquire about potential problems.

The study will be performed in accordance with the Good Clinical Practice (GCP) guidelines, the Declaration of Helsinki, the German Medical Drug Law and Data Protection Laws in their current or effective versions. An extensive GCP monitoring will be conducted. Additionally, we put strategies in place to maximize data quality, such as intensive training of the study team, nursing personal and parents. Adverse event management will be done according to standard regulations.

### Safety

The overall risk of the study is considered to be minimal. In the verum group, administration of carbogen will only reconstitute the normal physiological state. According to the laws of respiratory physiology it is impossible to increase the arterial pCO_2_ to toxic levels with 3 liter carbogen. Hence, the study-related pCO_2_-elevation is only short and normalizes within 30 seconds after termination of the gas application. The short-term oxygen application does not bear any risks and even improves the oxygenation of the patient during the seizure. Finally, we investigated in an adult volunteer what would happen if the gas flow ceased while the parents would continue to hold the mask in front of the child’s face. The experiment was continued for 6 minutes. After 3 minutes a small re-breathing effect was observed with elevation of the inspiratory pCO_2_ by 1-2 mmHg, but no elevation of endtidal pCO_2_. The oxygen saturation was always >97%. Since the breathing mask is only loosely fitting, ambient air would have enough access to prevent suffocation.

Definition, documentation and reporting of adverse and serious adverse events follow established conventions and regulatory requirements. The entire trial will be terminated prematurely in the event that more than 30% of the patients terminate their participation prematurely for the following individual reasons: **(i)** retraction of informed consent, **(ii)** subsequent occurrence one or more exclusion criteria, **(iii)** occurrence of a suspected unexpected serious adverse reaction (SUSAR), **(iv)** non-authorized use of the study medication as well as non-compliance of the parents or custodians of the study participant. Furthermore, the study will be terminated in the event that the risk-benefit-ratio changes towards a higher risk. Sealed unblinding envelopes are available at the study centre. Unblinding for any reason inevitably results in the exclusion of the patient. Parents are thoroughly trained by the study physician about what to do during a seizure or an emergency situation and when to call an ambulance to present to the Accident and Emergency Department of the Children’s University Hospital of the Charité, Berlin. The study team can be reached by mobile phone around the clock.

### Statistical analyses

Statistical planning is based on the modified intention-to-treat principle, i.e. only patients suffering from a febrile seizure recurrence during the study period of 24 months will be assigned to the intention-to-treat population. Analyses will be performed using SPSS, R, and SAS in their newest versions.

The aim of the study is the proof of the superiority of the experimental intervention (carbogen) *versus* control (oxygen) to suppress a seizure recurrence within 3 minutes. Success rates under intervention and control being p_I_ and p_K_, the statistical null hypothesis is p_I_ = p_K,_ and the alternative hypothesis p_I_ ≠ p_K_. The analyses will be carried out according to Bauer & Köhne with α = 0.025, c_α_= 0.00380 and α_0_ = 0.5 (in each case one-sided) [29]. The main variable “diazepam application “no” ( = success) *versus* “yes” (= failure)” will be analyzed as a binary endpoint by means of the Fisher´s exact test. The secondary endpoints safety and practicability will be analyzed by relative frequency and two-sided 95% confidence interval. The other secondary endpoints quality of life, anxiousness, mobility and contentedness will be analyzed in a descriptive manner according to their scaling; p-values will be reported but are not to be interpreted in a confirmatory way. The dependency of the repeated measures (“crossover” and “open label” phase) will be adjusted for by means of generalized estimating equation for a logistic regression model.

One-sided significance level will be 0.025. With an expected difference of 75% *versus* 25% the power for the two-sided Fisher´s exact test will be 86% when including 2 x 26 patients. The effective power will probably be even higher, as the sample size can only be increased but not decreased. After 2 x 10 patients with at least one seizure recurrence have been included, all data of those patients will be unblinded and will be analyzed with respect to safety. Furthermore, an interim analysis will be performed to adjust the sample size. A medical expert will decide whether the safety data allow the continuation of the study. If the study is continued, the new sample sized will be defined by means of the Bauer & Köhne method [29].

### Ethics

Following the legal requirements, no patient will be deprived of standard care due to study participation [34]. The study medication is applied during a time window of 3 minutes, at which the parents are advised, according to the guidelines, to wait before application of rectal diazepam (standard care). If the seizures persists even after application of diazepam parents are advised to call the emergency number, as it is the case in standard care. The study has been reviewed and approved by the Ethical Review Board of the State of Berlin.

The scheme for recruitment, randomization, and crossover of the CARDIF trial is depicted in the Flow diagram at Figure 4.

## Discussion

The CARDIF study will provide important data on a treatment alternative for patients with febrile seizures recurrences. This closes the gap between promising preclinical observations and so far missing evidence-based data on the efficacy and safety of such an approach. Possibly, the use of benzodiazepines might become dispensable or at least second choice behind the application of carbogen, which has fewer side effects. To our knowledge, CARDIF is presently the only registered clinical trial that investigates a new treatment to suppress an acute recurrence of a febrile seizure. Since this study is a non-commercial investigator-initiated trial mainly based on own preclinical and clinical data, it is a nice example for an academic translational approach. Some challenges and limitations of the CARDIF study may deserve a closer discussion.

Because febrile seizures only occur in children, the study hypothesis can only be tested in minors. All legal and ethical criteria specific for the conduct of clinical trials in minors (e.g. to be of benefit for the entire group of affected patients and to expose the study participants to the lowest possible risk and burden) had to be fulfilled by the study design to obtain regulatory approval.

Before initiating the CARDIF trial we attempted in another study to treat patients with a febrile seizure using a re-breathing device in a controlled in-patient setting. As febrile seizures are short unpredicted events and mainly occur at home, we were unable to treat more than 2 patients over a period of 12 months. As re-breathing from a plastic bag without safety monitoring is potentially dangerous, such approach must not be transferred into a domestic environment.

With this in mind we looked for an alternative and identified the low-pressure cans, which could be used to apply the desired amount of carbogen at home. The limited volume of gas prevents overdosage and the oxygen offers the added benefit to prevent hypoxia during the seizure. The main challenge resulting from this change of strategy was to find a company, which would be willing and certified to produces such cans with mask containing the gas in “medical quality” according to the requirements of the German Medical Drug Law.

A possible difficulty of the study results from the domestic use of the study medication. Hence, the primary outcome will not be assessed by the investigators but by medical lay persons (here the parents). Thus, the investigators have to rely on the compliance and reliability of these individuals with regard to correct application of the gas as well as the reporting of the outcome. Taking this into account, parents will receive intensive hands-on training and supervision, and will be contacted or visited regularly.

Only approximately 25-30% of children with a life history of one febrile seizure have further ones. Accordingly, thrice as many patients have to be recruited to the study than needed for the analysis. This includes the informed consent procedure, randomization, distribution of the study medication, and training. This will lead predictably to a high percentage of non-treated patients and non-used study medication.

In conclusion, the CARDIF study represents a prime example of an intramural translational approach of an academic institution: researchers from one research consortium working hand-in-hand with clinicians to translate their findings from basic science leading to novel therapies. This is made possible by the NeuroCure consortium at the Charité that offers an interdisciplinary forum, training for physician-scientists, protected research time for clinicians, as well as intramural funding for investigator initiated trials in order to overcome the common “translational roadblocks” [35,36].

## Abbreviations

CARDIF: CARbon DIoxide against Febrile seizures; CO2: Carbon dioxide; O2: Oxygen; pCO2: Partial pressure of CO_2_.

## Competing interests

The authors do not report any conflict of interest.

## Authors’ contributions

Design of the study, draft of the study protocol (SO, DS, MS), organization of all regulatory affairs (SO), draft of the manuscript (SO, MS), biometrical and biostatistical planning (PM), performing the respiratory investigations with the carbogen cans (MS, SWC, TW), acquisition of funding (DS, MS), critically revising the manuscript for intellectual content (SO, TW, SWC, CW, PM, DS, MS). All authors have given final approval of the version to be published.
